# CRISPR technology is revolutionizing the improvement of tomato and other fruit crops

**DOI:** 10.1038/s41438-019-0159-x

**Published:** 2019-06-15

**Authors:** Tian Wang, Hongyan Zhang, Hongliang Zhu

**Affiliations:** 1 College of Food Science and Nutritional Engineering, China Agricultural University, 100083 Beijing, China; 2 Key Laboratory of Food Nutrition and Safety of Shandong Normal University, College of Life Science, Shandong Normal University, 250014 Jinan, China; 3 College of Food Science and Nutritional Engineering, China Agricultural University, 100083 Beijing, China

**Keywords:** Genetic engineering, Molecular engineering in plants

## Abstract

Fruits are major sources of essential nutrients and serve as staple foods in some areas of the world. The increasing human population and changes in climate experienced worldwide make it urgent to the production of fruit crops with high yield and enhanced adaptation to the environment, for which conventional breeding is unlikely to meet the demand. Fortunately, clustered regularly interspaced short palindromic repeat (CRISPR) technology paves the way toward a new horizon for fruit crop improvement and consequently revolutionizes plant breeding. In this review, the mechanism and optimization of the CRISPR system and its application to fruit crops, including resistance to biotic and abiotic stresses, fruit quality improvement, and domestication are highlighted. Controversies and future perspectives are discussed as well.

## Introduction

Fruits are major sources of fibers, vitamins, and minerals worldwide^1^. In some parts of Asia, Africa, and South America, banana, breadfruit, and date fruit also serve as staple foods^2–4^. Fruit crops are at high risk under climate change^5^. To increase the chances of a steady fruit supply, our ancestors domesticated wild plant species into cultivated crops. Following the “rediscovery” of Mendel’s laws in 1900, breeders started selecting and crossing superior plants^6^. However, conventional breeding has major shortcomings. First, it largely depends on existing natural allelic variations and is thus inefficient for obtaining the desired characteristics by random mixing of tens of thousands of genes^5^. Although conventional breeding has increased crop productivity, it is often accompanied by loss of fitness and genetic diversity^7^, and it is a rather time-consuming practice that could hardly ensure a sufficient food supply for the rapidly growing human population around the world^8^. Therefore, continuous technological innovation is required to meet the increasing demands of consumers^9^.

Genetic engineering techniques have numerous applications in fruit crops, as they allow improvement of important agronomic traits such as biotic and abiotic stress tolerance and fruit quality. During the past two decades, several fruit crops have been modified using these techniques. In contrast to conventional breeding, recombinant DNA technology allows transfer of the desired genes from any organism, plant or microorganism into fruit crops, extending the opportunities for fruit yield enhancement by offering new genotypes and phenotypes for breeding purposes, and ultimately improving fruit quality as well as enhancing shelf life. Thus, genetic engineering has been ranked as the fastest developing technology in agriculture^10^. The organisms obtained by recombinant DNA technology are termed “genetically modified” (GM). In 1994, the transgenic “Flavr Savr tomato” was approved for commercial growth in the United States (US) by the Food and Drug Administration (FDA). The modification it contained allowed a slowing of its ripening process and prevented it from softening after picking. The GM papaya authorized for marketing can resist ring spot virus attacks and show enhanced productivity. Eighty percent of Hawaiian papaya produced today is genetically engineered, and no alternative method is available^11^.

However, the development of new GM crops is largely affected by regulatory-approval processes because the purpose of the approval system is preventing harm to human health and the environment, as well as avoiding economic losses^12^. These regulations also help ensure consumer confidence in GM crop biosafety^13^. As a result, the costs of obtaining approval for new GM crops can be very high, and the regulatory requirements may also delay product marketing^14^. Jefferson et al.^15^ have argued that these stringent regulations can result in unnecessary barriers to the introduction of new GM crops. Thus, clustered regularly interspaced short palindromic repeat (CRISPR) technology may be a better choice: in 2016, a CRISPR-edited mushroom escaped US regulation as it fell outside the GM organisms legislation by not containing foreign DNA^16^. In 2017, the FDA approved the marketing of a false flax with increased oil content and a drought-tolerant soybean^17^, indicating that the CRISPR-edited crops were not under the same stringent regulations as traditional GM crops and that the CRISPR technology would definitely revolutionize the pace of crop breeding^18^.

## Genome editing has been revolutionized by the development of CRISPR technology

### The discovery of CRISPR in the prokaryote immune system

The CRISPR system is a sophisticated adaptive immune mechanism present in bacteria and Archaea for defense against invading bacteriophages and exogenous plasmids^19^. It was first discovered in the genome of *Escherichia coli* in 1987^20^ and officially named by the Dutch scientist who identified *CRISPR-associated* (*Cas*) genes^21^. In 2005, three different research groups simultaneously found that the short sequences of many CRISPR spacers were highly homologous with sequences originating from extra chromosomal DNA^22–24^, indicating a relationship between CRISPR and specific immunity. Nearly a decade later, CRISPR-Cas was successfully engineered into an efficient tool to edit human, animal, and plant genomes^25,26^, extensively boosting its application in fields as diverse as pharmacology, animal domestication, and food science^27^.

A complete CRISPR-Cas locus comprises a CRISPR array that harbors short repetitive elements intercalated with invader DNA-targeting spacers, an AT-rich leader sequence, and an operon of *Cas* genes encoding the Cas proteins^28^. Based on the different participating Cas proteins, CRISPR-Cas systems can be categorized into three main types: type I and type III systems use a large multi-Cas protein complex for binding and targeting^29,30^, while the type II system requires only a single protein, the CRISPR-associated protein 9 (Cas9), for RNA-guided double-stranded DNA recognition and cleavage using its two distinct domains, RuvC and HNH^31^. The simplicity of the type II CRISPR (i.e., of the CRISPR-Cas9 system) enabled remarkable progress in genome engineering^32^.

### The mechanism of CRISPR-Cas9

In general, the action of the CRISPR-Cas9 system can be divided into three stages in response to invading foreign DNA^33,34^: (i) acquisition stage—the invading DNA is identified and a spacer sequence derived from the target DNA is inserted into the host CRISPR array to establish immunological memory; (ii) expression stage—the Cas9 protein is expressed, and the CRISPR array is transcribed into a precursor RNA transcript (pre-crRNA). A non-coding trans-activating CRISPR RNA (crRNA) then hybridizes to the pre-crRNA and Cas9 protein and processes them into mature RNA units known as crRNAs; and (iii) interference stage—the mature crRNA guides the Cas9 protein to recognize the appropriate DNA target, leading to the cleavage and degradation of the invading foreign DNA.

**Fig. 1:**
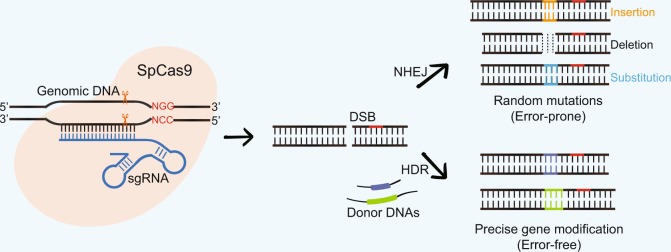
The mechanism of CRISPR-Cas9-mediated genome engineering in plants. The sgRNA directs the SpCas9 protein to bind genomic DNA through a 20-nucleotide sequence and further guides it to introduce a DSB. This DSB causes random mutations when repaired by the error-prone NHEJ pathway or precise gene modification when repaired by the error-free HDR pathway. CRISPR, clustered regularly interspaced short palindromic repeat; Cas, CRISPR-associated; DSB, double-strand break; HDR, homology-directed repair; NHEJ, non-homologous end-joining; sgRNA, single-guide RNA

The Cas9 protein cuts the DNA to generate a double-strand break (DSB), triggering cellular DNA repair mechanisms (Fig. 1). In the absence of a homologous repair template, the error-prone non-homologous end-joining (NHEJ) pathway is activated and introduces random insertions/deletions or even substitutions at the DSB site, generally resulting in the disruption of gene function. Alternatively, if donor DNA template homologous to the sequence surrounding the DSB site is available, the error-free homology-directed repair (HDR) pathway is initiated, leading to mutations that perform precise gene modification, including gene knock-in, deletion, or mutation^35^. At present, the most commonly used Cas9 protein comes from *Streptococcus pyogenes* (*Sp*)^36^. To exploit this system for genome editing, synthetic single-guide RNAs (sgRNAs) are required to construct the CRISPR-Cas9 expression cassettes. The Cas9 protein is then guided to specific genomic sites by the sgRNAs that recognize the NGG-type protospacer adjacent motif and targets DNA sequences through Watson–Crick base pairing^37^ (Fig. 1).

### The optimization of the CRISPR-Cas system in plants

Since the CRISPR-Cas system was successfully engineered to edit plant genomes in 2013, numerous efforts have been made to transform it into a more powerful tool. At present, CRISPR-Cas has multiplex editing capability, that is, it edits more than one gene at a time^38^. In addition, CRISPR-Cas can target not only the open reading frame (ORF)^39^ and untranslated region^40^ of one coding gene but also non-coding RNAs (ncRNAs) including long ncRNA^41^ and microRNA^42^, as well as promoter regions^43^. Single-base substitutions at genomic targets without requiring DSBs have also been achieved^44^. Here, we describe the optimization of the CRISPR-Cas system regarding the diversified development of Cas proteins, the optimization of Cas promoters, and the empowerment of sgRNAs with multiplexing capability (Table 1).

**Table 1:** Optimization of the CRISPR-Cas system in plants

Name	From	Function	Crop species	Refs.
Cas proteins				
St1Cas9	*Streptococcus thermophilus*	Size is smaller; recognizes longer PAMs (“NNAGAA” or “NNGGAA”)	*Arabidopsis*	45
SaCas9	*Staphylococcus aureus*	Size is smaller; recognizes longer PAMs (“NNGGGT” or “NNGAA”)	*Arabidopsis*; tobacco	45,46
SpCas9-VQR	*Streptococcus pyogenes*	Recognizes “NGA” PAM	Rice	47
SpCas9- VRER	*Streptococcus pyogenes*	Recognizes “NGCG” PAM	Rice	47
Cas12a (Cpf1)	*Acidaminococcus* sp. *BV3L6* (*As*); *Francisellanovicida* (*Fn*); *Lachnospiraceae* bacterium ND2006 (*Lb*)	Recognizes “TTTN” or “TTN” PAMs; targets DNA to introduce a 5′ overhang; guided by a shorter crRNA; exhibits little off-target activity	*Arabidopsis*; maize; rice; soybean; tobacco	48–51
Cas13a (C2c2)	*Leptotrichiashahii*	Targets single-stranded RNA with PFS of A, U, or C	Rice; tobacco	52,53
nCas9	*Streptococcus pyogenes*	Cas9 nickase contains a mutation in either of the two nuclease domains of Cas9 protein. It induces SSBs	*Arabidopsis*; rice; tomato	54–56
dCas9	*Streptococcus pyogenes*	Deficient Cas9 contains mutations in both nuclease domains of Cas9 protein. without cleavage activity. The dCas9-based regulator can be developed when fused with transcriptional activators or repressors	*Arabidopsis*; maize; rice; tobacco; wheat	56–59
Promoters		Preferential expression	Crop species	Refs.
Cas promoters				
* YAO*		Tissues undergoing active cell division including the shoot apical and root meristem, embryo sac, embryo, endosperm, and pollen	*Arabidopsis*; citrus	60,61
* SPL*		Sporogenous cells and microsporocytes	*Arabidopsis*	62
* EC1.1/EC1.2*		Egg cells and one-cell stage embryos	*Arabidopsis*	63,64
* ICU2*		Meristematic regions	*Arabidopsis*	65
* EF1α*, *hisH4*		Meristematic and reproductive tissues	*Arabidopsis*	66
* MGE*		Meiosis stage	*Arabidopsis*	67
* DMC1*		Meiocytes	*Arabidopsis*; maize	68,69
* RPS5A*		At all developmental stages	*Arabidopsis*	70
Strategy			Crop species	Refs.
sgRNAs				
Assemble multiple sgRNA expression cassettes into CRISPR-Cas vector			*Arabidopsis*; maize; Populus; rice; tobacco; tomato	71–75
Produce numerous sgRNAs from a single polycistronic gene via the endogenous tRNA-processing system			Maize; potato; rice; tomato; wheat	76–80

*PAM* protospacer adjacent motif, *sgRNA* single-guide RNA, *CRISPR-Cas* clustered regularly interspaced short palindromic repeat-CRISPR-associated, *tRNA* transfer RNA, *PFS* protospacer flanking sequence, *SSBs* single-strand breaks, *crRNA* CRISPR RNA

## Applications of CRISPR-Cas9 in fruit crops

**Fig. 2:**
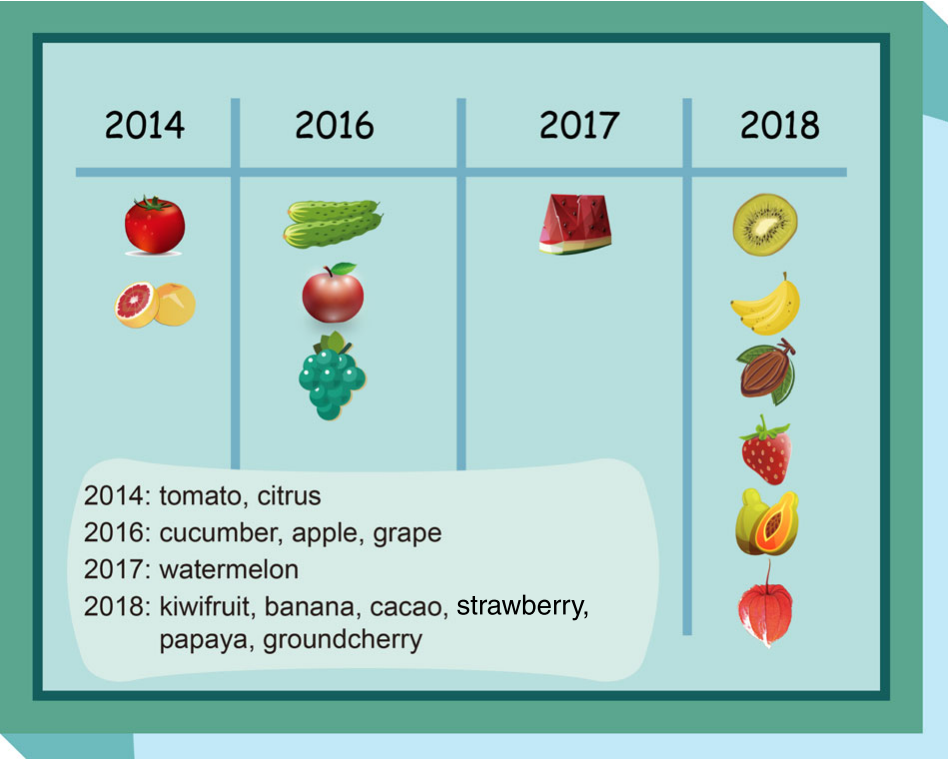
Timeline of the first application of the clustered regularly interspaced short palindromic repeat-CRISPR-associated (CRISPR-Cas9) system in fruit crops

Duane Green has defined a fruit crop as a perennial, edible crop where the economic product is the true botanical fruit or derived from it^81^. Some plants, grown primarily as annuals, such as tomatoes, cucumbers, and melons, are also considered fruit crops^82^. Due to its easily achieved germplasm resources, simple diploid inheritance, efficient breeding, short growing period, ease of genetic transformation, and extensive research, tomato acts as a model for fruit biology^1^. Here, we summarize the applications of the CRISPR-Cas9 system in tomato and other fruit crops (Fig. 2 and Table 2).

**Table 2:** Current applications of CRISPR-Cas9 in fruit crops

Crop species	Target genes	Target traits	Refs.
Resistance to biotic stresses			
Tomato	*CP* and *Rep* of virus	Resistance against tomato yellow leaf curl virus	83
Tomato	*DCL2*	Susceptibility to potato virus X, tobacco mosaic virus, and tomato mosaic virus	84,85
Tomato	*DMR6*	Resistance against downy mildew	86
Tomato	*MLO1*	Resistance against powdery mildew	87
Tomato	*PMR4*	Resistance against powdery mildew	88
Tomato	*Solyc08g075770*	Susceptibility to *Fusarium* wilt disease	89
Tomato	*MAPK3*	Susceptibility to gray mold disease	90
Tomato	*JAZ2*	Resistance against bacterial speck disease	91
Banana	ORF region of virus	Resistance against banana streak virus	92
Cucumber	*eIF4E*	Resistance against cucumber vein yellowing virus, zucchini yellow mosaic virus, and papaya ring spot mosaic virus	93
Grape	*MLO7*	Resistance against powdery mildew	94
Grape	*WRKY52*	Resistance against gray mold disease	95
Cacao	*NPR3*	Resistance against *Phytophthora tropicalis*	96
Papaya	*alEPIC8*	Resistance against *Phytophthora palmivora*	97
Citrus	*LOB1 promoter*	Resistance against citrus canker	98,99
Apple	*DIPM1, 2, 4*	Resistance against fire blight disease	94
Resistance to abiotic stresses			
Tomato	*BZR1*	Decrease in heat stress tolerance	100
Tomato	*CBF1*	Decrease in chilling stress tolerance	101
Tomato	*MAPK3*	Decrease in drought stress tolerance	102
Watermelon	*ALS*	Resistance against herbicide	103
Fruit quality improvement			
Tomato	*CLV3, lc*	Fruits with increasing locule numbers	104
Tomato	*PSY1*	Yellow-colored tomato	105
Tomato	*MYB12*	Pink-colored tomato	106
Tomato	*ANT2* (gene insertion)	Purple-colored tomato	107
Tomato	*PL*	Long-shelf life tomato	108
Tomato	*ALC*	Long-shelf life tomato	109
Tomato	*MPK20*	Repression of genes controlling sugar metabolism	110
Tomato	*ANT2* (gene insertion)	Increase in anthocyanin content	107
Tomato	*GAD2*, *GAD3*	Increase in GABA content	111
Tomato	*GABA-TP1*, *GABA-TP2*, *GABA-TP3*, *CAT9*, *SSADH*	Increase in GABA content	112
Tomato	*SGR1, LCY-E, Blc, LCY-B1, LCY-B2*	Increase in lycopene content	113
Tomato	*ALMT9*	Decrease in malate content	114
Fruit crop domestication			
Tomato	*AGL6*	Production of parthenocarpic fruit	115
Tomato	*IAA9*	Production of parthenocarpic fruit	116
Tomato	*ARF7*	Production of parthenocarpic fruit	117
Tomato	*MBP21*	Generation of “jointless” fruit stem	118
Tomato	*GAI*	Generation of dwarf tomato plants	119
Tomato	*BOP1, BOP2, BOP3*	Early flowering with simplified inflorescences	120
Tomato	*SP, SP5G, CLV3, WUS, GGP1*	Introduction of traits associated with morphology, flower and fruit production, and ascorbic acid synthesis	121
Tomato	*SP, OVATE, MULT, FAS, CycB*	Introduction of traits associated with morphology, flower number, tomato size and number, and lycopene synthesis	122
Tomato	*SP5G*	Generation of loss of day-length-sensitive tomato plants	123
Cucumber	*WIP1*	Generation of gynoecious plant	124
Groundcherry	*SP, SP5G, CLV1*	Introduction of traits associated with morphology, flower production, and fruit size	125
Kiwifruit	*CEN4, CEN*	Generation of a compact plant with rapid terminal flower and fruit development	126

*CRISPR-Cas* clustered regularly interspaced short palindromic repeat-CRISPR-associated, *ORF* open reading frame, *GABA* γ-aminobutyric acid

### Current applications of CRISPR-Cas9 in tomato

In 2014, the CRISPR-Cas9 system was first applied in tomato. *Argonaute 7* was knocked out resulting in wiry phenotypes; the first leaves of mutants had leaflets without petioles and subsequently formed leaves lacking laminae^127^. Since then, numerous publications on CRISPR-Cas9 application in tomato have been published. We classified these publications into the following four groups: resistance to biotic stresses, resistance to abiotic stresses, improvement of tomato fruit quality, and domestication of tomato.

#### Resistance to biotic stresses

Biotic stresses include viruses, bacteria, fungi, and insects, all of which can attack plants and cause damage^128^. CRISPR-Cas9 technology has been employed to obtain disease-resistant plants^129^ since its successful application for obtaining stable transgenic lines in 2013. Since then, CRISPR-Cas9 has been used against viral, fungal, and bacterial infection, which causes severe losses in tomato^130,131^.

For viruses, two strategies have been used. One consists of designing sgRNAs and targeting the virus genome directly through sequence complementation, and the other consists of modifying the tomato genes that confer antiviral characteristics. Tashkandi et al.^83^ used the CRISPR-Cas9 system to engineer tomato plants resistant to the tomato yellow leaf curl virus by targeting the coat protein and replicase loci of the genome. The transgenic tomato showed efficient viral interference and accumulated less viral genomic DNA than the wild-type (WT) plants. This kind of immunity remained active across multiple generations, indicating the utility of the CRISPR-Cas9 system for cultivating durable virus resistance plants. CRISPR-Cas9 technology has also been used to knock out crucial genes involved in resistance pathways, aiming to test whether these genes can confer immunity against viruses. Tomato *Dicer-like 2* (*DCL2*) genes were targeted, and the *dcl2* mutants displayed viral symptoms when infected by potato virus X, tobacco mosaic virus, and tomato mosaic virus, suggesting that *DCL2* is involved in the defense mechanism against RNA viruses^84,85^.

Fungi are accountable for multiple diseases, including mildew, smut, rust, and rot, which can cause dramatic losses in crop yield and quality^130^. Downy and powdery mildews inflict severe economic losses in tomato. In *Arabidopsis thaliana*, *downy mildew resistant 6* (*DMR6*), which belongs to the 2-oxoglutarate Fe(II)-dependent oxygenase superfamily, participates in salicylic acid homeostasis, and its overexpression results in enhanced susceptibility to downy mildew^132^. Researchers have used the CRISPR-Cas9 system to inactivate the *DMR6* ortholog in tomato and found that *dmr6* mutants showed disease resistance against various pathogens, including *Pseudomonas syringae*, *Phytophthora capsica*, and *Xanthomonas* spp., without significant detrimental effects^86^. *Mildew resistant locus O 1* (*Mlo1*), which encodes a membrane-associated protein, confers susceptibility to the fungi causing powdery mildew disease. Nekrasov et al.^87^ generated the tomato loss-of-function *mlo1* mutant using CRISPR-Cas9 technology and found that the mutant was fully resistant to the powdery mildew fungus *Oidium neolycopersici*. Notably, the authors segregated the transfer DNA (T-DNA) by selfing T0 transformants, and among the progeny, they identified *mlo1* T-DNA-free mutants, which were regarded as transgene-free crops^87^. *Powdery mildew resistance 4* (*PMR4*), which encodes a callose synthase, also leads to resistance against *O. neolycopersici*^88^. Another well-known tomato fungal pathosystem is *Fusarium oxysporum*^131^, which can cause *Fusarium* wilt disease. The yield of tomato fruit is negligible in highly infected plants. The *Solyc08g075770 gene* has been identified to function in *Fusarium* wilt tolerance, and CRISPR-Cas9 knockout transgenic plants exhibited disease susceptibility^89^. *Botrytis cinerea* is an airborne plant pathogen that causes gray mold disease, resulting in serious economic losses in both pre- and postharvest stages. Tomato is susceptible to postharvest infection by *B. cinerea*^133^. Mitogen-activated protein kinase 3 (MAPK3) has been shown to confer resistance to *B. cinerea* by using CRISPR-Cas9 technology^90^.

Due to undetectable asymptomatic infections and a lack of suitable agricultural chemicals, plant pathogenic bacteria are hard to control, and using genetic resistance against these pathogens is the most efficient strategy^130^. *Pseudomonas syringae* is the causative agent of the bacterial speck disease in tomato plants, negatively affecting their productivity and marketability. Because *Jasmonatezim domain protein 2* (*JAZ2*) contributes to the defense against *P. syringae* in *A. thaliana*^134^, researchers used CRISPR-Cas9 to generate tomato dominant JAZ2 repressors lacking the C-terminal jasmonate associated (Jas) domain (JAZ2Δjas). These *JAZ2Δjas* repressors provide resistance to *P. syringae*, indicating that a CRISPR-Cas9-based strategy for fruit crop protection can be implemented in the field^91^.

#### Resistance to abiotic stresses

According to Charles Darwin's evolutionary theory, it is not the most intellectual or strongest species that survives, but the one that is able to adapt to and adjust best to the changing environment in which it finds itself^135^. Abiotic stresses such as drought, flooding, heat, and chilling, especially those under a climate change scenario, pose high risks to species, especially crops^136^. Traditional breeding techniques have greatly increased crop yield, but with the growing demand for food, new approaches are needed to further improve crop production, and CRISPR-Cas9 technology is the most promising^137^.

*Brassinazole resistant 1* (*BZR1*) regulates brassinosteroid (BR) response and participates in BR-mediated developmental processes. Its ortholog in tomato also controls BR response. *BZR1* is also involved in thermotolerance by regulating the *Feronia* (*FER*) genes, as verified by both CRISPR-*bzr1*- and *BZR1*-overexpressing lines^100^. Because tomato is a chilling-sensitive crop, its fruit quality is easily damaged due to chilling stress. Li et al.^101^ found that *C-repeat binding factor 1* (*CBF1*) protects plants from cold injury, as the *cbf1* mutant generated by CRISPR-Cas9 exhibited more severe chilling-injury symptoms with higher electrolyte leakage than WT plants. *MAPK3*, which participates in resistance against gray mold disease^90^, is also involved in tomato drought response by protecting cell membranes from oxidative damage^102^.

#### Improvement of tomato fruit quality

Fruit quality can be defined based on external and internal characteristics. The external quality factors are fruit size, color, and texture, all easily detected with the naked eye. Internal fruit quality attributes, including the levels of nutrients (such as sugar and vitamin) and bioactive compounds (such as lycopene, anthocyanin, and malate), need to be measured by instruments^138^.

In tomato fruit, the number of locules derived from the flower carpels has the greatest effect on tomato fruit size, contributing as much as 50% to the total variance in fruit enlargement. Locule number is controlled by multiple quantitative trait loci (QTL), a few of which have been identified^139^. Scientists at the Cold Spring Harbor Laboratory used CRISPR-Cas9 technology to rapidly generate larger tomato fruits by destructing the classical *CLAVATA-WUSCHEL* (*CLV-WUS*) stem cell circuit^140^. Eight sgRNAs were designed to target the promoter region of the *CLV3* gene, and transgenic plants produced more organs and larger fruits than WT plants. The researchers also recreated a known fruit size QTL, *locule number* (*lc*) in tomato, generating fruits with increasing locule number^104^. Color and texture are also important aspects of consumer perception of fresh tomatoes^141^. Consumers from different areas have different color preferences. For instance, European and American consumers prefer red tomatoes, while in Asia, pink-colored tomatoes are more popular^142,143^. Researchers have successfully cultivated yellow^105^, pink^106^, and purple^107^ tomatoes by targeting *phytoene synthase 1* (*PSY1*), *MYB transcription factor 12* (*MYB12*), and *Anthocyanin 2* (*ANT2*), respectively. Modifying texture characteristics for a prolonged shelf life has long been a challenge for breeders. The inactivation of *ripening inhibitor* (*RIN*) or *DNA demethylase 2* (*DML2*) by CRISPR can lead to incomplete ripening fruits with long shelf life^144,145^. However, these fruits usually fail to develop full color, resulting in poor flavor and reduced nutritional value. Hence, obtaining fruits that exhibit good shelf life without affecting other quality aspects is crucial. Two research groups have reported successful harnessing of fruit softening by silencing *pectate lyase* (*PL*) and *alcobaca* (*ALC*) without reducing tomato organoleptic and nutritional quality^108,109^, suggesting that the CRISPR system might be an excellent tool for fruit crop improvement.

Regarding internal fruit quality, much effort has been made to increase the levels of nutrients and bioactive compounds. Carbohydrates and vitamins are vital nutrients because they provide energy. Several genes are involved in the synthesis and metabolism of sugar and carotenoids (provitamin A carotenoid can be absorbed and converted to vitamin A in the human body). For example, knocking out *mitogen-activated protein kinase 20* (*MPK20*) disrupted the expression of several genes that control sugar metabolism at both the transcript and protein levels^110^. Bioactive compounds are defined as “extra nutritional constituents that typically occur in small quantities in foods” and usually play roles in the prevention of cardiovascular disease and cancer^146^. Anthocyanin^147^, malate^114^, γ-aminobutyric acid (GABA)^111^, and lycopene^113^ are considered bioactive compounds, and CRISPR-Cas9 technology has been applied to produce anthocyanin-, GABA- and lycopene-enhanced tomato fruits by modulating the expression of key genes in their metabolic pathways^107,111–113^. The key gene that determines tomato malate content, *aluminum-activated malate transporter 9* (*ALMT9*), has also been identified using CRISPR-Cas9^114^.

#### Domestication of tomato

Domestication of plants mostly affects the genes controlling plant morphology (seed size, dispersal mechanism, and plant architecture) and physiology (timing of germination, flowering, and ripening)^148,149^. To achieve the ideotype, classical breeding or modern “rewilding” crop breeding have introduced alleles from wild relatives into cultivated species. However, these techniques are time-consuming. An alternative strategy is direct manipulation of wild crops at the gene level to domesticate them de novo and harness their adaptation to adverse environments^150^. This de novo domestication has been substantially accelerated by the CRISPR-Cas9 technology.

Parthenocarpy, a fertilization-independent seedless fruit development, is regarded as a desirable agronomic trait in fruit crops: (i) it is advantageous for stable crop yield in fluctuating environments; (ii) it saves energy when separating the seeds from processed products for industrial purposes; and (iii) consumers prefer seedless over seeded fruits^115–117^. Klap et al.^115^ confirmed that a mutation in *agamous-like 6* (*AGL6*) is responsible for parthenocarpic fruit production under heat stress conditions; because the mutant is of normal weight and shape, without homeotic changes, *AGL6* is an attractive gene for parthenocarpy. Elevated gibberellin or auxin signaling can induce parthenocarpy without fertilization. The mutants produced by the knock out of *indole-3-acetic acid inducible 9* (*IAA9*) and *auxin response factor 7* (*ARF7*), both involved in the auxin signaling pathway, produced seedless fruits, which is a characteristic of parthenocarpic tomato^116,117^. The joint is a weak region of the stem that allows the fruit to drop from the plant. Wild species benefit from dropping fruit because this process contributes to seed dispersal, but because they use picking manipulators, farmers prefer to have fruit hanging on the plant. Breeders have been trying to obtain a mutant that eliminates the flower abscission zone (by which unfertilized flowers or ripe fruit are shed from the plant) and provides a “jointless” fruit stem^151,152^. Roldan et al.^118^ developed the *MADS-box protein 21* (*MBP21*) loss-of-function mutant *mbp21* exhibiting the jointless phenotype using CRISPR-Cas9 technology^118^. Fruits are easier to pick, and nutrients are transported over shorter distances from the roots to the leaves in dwarf plants compared with normal plants. Dwarf plants are also more likely to survive when exposed to strong winds. Heritable dwarf tomato plants have been generated by inactivating the *gibberellic-acid insensitive* (*GAI*) gene, and these plants can be useful in windy environments. However, the reduced fruit weight and seed number issues of these dwarf mutants need to be solved first^119^. Plant productivity depends on flowers, and inflorescence architecture determines flower production. CRISPR-Cas9 technology was used to silence the tomato *blade-on-petiole* (*BOP*) gene to test whether it has the same function as its homolog in *A. thaliana* (leaf complexity and organ abscission), which affects inflorescence architecture. Notably, the *CRISPR-bop1/2/3* triple mutant flowered faster than the WT, but with extremely simplified inflorescences^120^.

Domestication of wild tomato species for commercial cultivation usually requires numerous phenotypes to be changed, including fruit setting and size, ripening synchrony, flowering and day-length sensitivity, and nutrient content^121^. Two research groups have recently devised a CRISPR-Cas9 technology that combines agronomically desirable traits with useful traits present in wild lines. One group targeted six loci of five genes critical for the productivity of present tomato lines, enabling the de novo domestication of wild *Solanum pimpinellifolium* whose morphology was altered, together with the size, number, and nutritional value of its fruits^122^. The other group introduced desirable traits into *S. pimpinellifolium* by editing coding sequences, *cis-*regulatory regions, or upstream ORFs of genes associated with morphology, flower and fruit production, and ascorbic acid synthesis^121^.

Sensitivity to day-length limits the geographical distribution of crops. Therefore, modification of the photoperiod response can help accelerate crop domestication processes. The loss of the day-length-sensitive tomato mutant produced by knocking out *self-pruning 5G* (*SP5G*) showed a quick burst of flower production that translated into an early fruit yield^123^.

### Current applications of CRISPR-Cas9 in other fruit crops

The use of CRISPR-Cas9 technology is not limited to tomato. It has also been successfully applied to several other fruit crops, including strawberry^153^, banana^154^, grape^155^, apple^156^, watermelon^157^, and kiwifruit^158^. As a model organism, strawberry is often used for the functional analysis of specific genes. For instance, targeting *R2R3 MYB transcription factor 10* (*MYB10*) leads to the generation of loss-of-coloration fruits^159,160^. Zhou et al.^153^ used CRISPR-Cas9 to target *auxin response factor 8* (*ARF8*) and identified that *arf8* homozygous mutants show faster seedling growth than WT plants. The *tomato MADS-box gene 6* (*TM6*) is reported to play a predominant role in stamen development^161^. To characterize its function in strawberry, the CRISPR-Cas9 system was applied to an octoploid species, and the phenotypic analysis of *tm6* mutants revealed severe defects in their anthers, indicating that *TM6* played an essential role in flower development^162^. In addition, the CRISPR-Cas9 strategy was used to investigate the biological role of *YUCCA 10* (*YUC10*) in auxin synthesis during strawberry fruit development. When *YUC10* was knocked out, a significant reduction in free auxin was observed in *yuc10* mutants^163^. In addition to the functional study in strawberry, an increasing number of researchers are focusing on CRISPR-Cas9-mediated genome editing for improvement of other fruit crops. Here, we summarize the recent applications of CRISPR-Cas9 to other fruit crops considering the following aspects: resistance to biotic stresses, resistance to abiotic stresses, and domestication of fruit crops (Table 2).

#### Resistance to biotic stresses

In tropical and subtropical countries, the banana streak virus is a major challenge in banana breeding^92^. As mentioned above, one strategy for improving resistance to viruses is targeting their genomes with CRISPR-Cas9. Tripathi et al.^92^ used this system to inactivate the endogenous banana streak virus and found that 75% of the edited plants remained asymptomatic in comparison to the non-edited control. Plant RNA viruses require a host factor, such as the eukaryotic translation initiation factor 4E (eIF4E), to maintain their life cycle. If the factor is inactivated, viral infectivity is disrupted. A virus-resistant cucumber mutant was developed using CRISPR-Cas9 to disrupt the function of *eIF4E*. As expected, the *eif4e* mutant exhibited immunity to cucumber vein yellowing virus, zucchini yellow mosaic virus, and papaya ring spot mosaic virus^93^. Fungal diseases can cause drastic losses in grapevine yield and grape berry quality. Two genes, *mildew resistance locus O 7* (*MLO7*) and *WRKY transcription factor 52* (*WRKY52*), are known to be involved in *Erysiphe necator* and *B. cinerea* resistance, respectively. Two research groups validated the functions of these genes using CRISPR-Cas9. Both loss-of-function mutants showed increased immunity^94,95^. This technology was also used in cacao and papaya to increase resistance against *Phytophthora tropicalis* and *Phytophthora palmivora*^96,97^. Citrus canker, caused by *Xanthomonas citri*, is a severe disease among most commercial citrus cultivars and is responsible for substantial economic losses worldwide. Two recent publications^98,99^ have reported the use of CRISPR-Cas9 for generating citrus plants resistant to citrus canker by targeting the promoter region of the *lateral organ boundaries 1* (*LOB1*) gene in citrus; the mutated lines showed high degrees of resistance to *X. citri* infection. Similarly, in apple protoplasts, the genes encoding DspA/E-interacting proteins (*DIPM1*, *DIPM2*, and *DIPM4*) were knocked out to improve resistance against *Erwinia amylovora*^94^. Date palm is an important fruit crop in desert agriculture. Due to its large and complex genome and high frequency of single-nucleotide polymorphisms, the application of CRISPR-Cas9 is a challenging task, and therefore, few genetic improvement studies have been performed. However, Satter et al.^164^ presented a generalized stepwise and basic strategy for the theoretical implications of CRISPR-Cas9, addressing its potential applications in date palm.

#### Resistance to abiotic stresses

Field watermelons are severely threatened by weeds, but the use of herbicides also affects their growth. Therefore, herbicide-resistant watermelons should be obtained, which is difficult to achieve via traditional breeding. In recent years, CRISPR-mediated single-nucleotide conversion has been used to develop herbicide-resistant rice^56^. To introduce this new base-editing system in watermelon, Tian et al.^103^ selected *acetolactate synthase* (*ALS*), a gene in which point mutations confer a high level of herbicide resistance. The transgene-free *als* mutants and WT plants were treated with the herbicide tribenuron, and while all WT plants were severely damaged, the *als* mutants were not, suggesting the successful establishment of a CRISPR base-editing system and herbicide-resistant watermelons^103^.

#### Domestication of fruit crops

Gynoecious lines benefit cucumber breeding, as they allow earlier generation of hybrids, higher yield, and more concentrated fruit set; eliminate the requirement for artificial emasculation; and reduce the labor cost of crossing compared to monoecious lines. WIP domain-containing protein 1 (WIP1) inhibits carpel development in cucumber, and the loss-of-function *wip1* mutant displays a gynoecious phenotype, bearing only female flowers in upper nodes^124^. Lemmon et al.^125^ domesticated an orphan crop, groundcherry, a wild *Solanaceae* grown in Central and South America. Using CRISPR-Cas9, three orthologs of tomato (*self-pruning* (*SP*), *SP5G*, and *CLV1*) that control plant architecture, flower production, and fruit size, respectively, were introduced into groundcherry, thereby improving these major productive characters in this crop. This successful application will accelerate the domestication of orphan crops by introducing known agronomic traits from distantly related model crops^125^. Kiwifruit is a recently domesticated fruit crop with large potential for improvement. By inactivating *centroradialis 4* (*CEN4*) and *CEN*, which have been validated as repressors of flowering, the original climbing woody perennial was transformed into a compact plant with rapid terminal flower and fruit development^126^.

## Concluding remarks

CRISPR-Cas9 technology has revolutionized crop breeding since its first application in 2013. The major breakthroughs were the generation of disease-resistant and environment-adaptive fruit crops, as well as improvement of fruit quality. Notably, the DNA-free delivery of preassembled CRISPR-Cas9 ribonucleoproteins has been conducted in plant protoplasts of *A. thaliana*, rice, tobacco, lettuce, wheat, and potato^165–168^. Plants originating from this technology might be considered non-GM crops. This characterization would open the door for the development of fruit crops with superior phenotypes and allow their commercialization and marketing even in countries where GM crops are unacceptable^169^. In April 2016, the FDA indicated that the CRISPR-edited mushroom could enter the market without oversight, making it the first CRISPR-edited organism to receive such authorization from the US government^16,170^. In 2017, the FDA allowed the marketing of false flax, with enhanced omega-3 oil, and drought-tolerant soybean, clearly indicating that CRISPR-edited plants can be cultivated and sold free from regulation^17^ and thereby providing great confidence to research focusing on the application of CRISPR to fruit crops.

However, the growth of CRISPR-edited crops faces sociopolitical challenges, including public acceptance and government regulation^171^. Although transgene-free organisms edited by CRISPR-Cas9 are not currently regulated in the US, whether to govern the use of CRISPR technologies is still being discussed in China and Japan^172^. According to the decision of Europe’s highest court earlier in 2018, gene-edited crops should be subject to the same stringent regulations that govern conventional GM organisms, which is a major setback for proponents, including many scientists^173^. With further advances in CRISPR technology and the establishment of an evaluation system, more countries might be willing to foster an optimistic and inclusive attitude toward CRISPR-edited crops. As researchers, in addition to further investigating CRISPR technology to ensure maximum benefit while minimizing risks, we need to be concerned with public acceptance. Most importantly, the basic aspects of this technology need to be explained sufficiently well to facilitate rational public discourse, increasing public confidence in the safety and advantages of CRISPR-edited crops. Governments might then express a *laissez faire* attitude after gaining strong public trust.
